# Should I stay or should I go? Determinants of immediate and delayed movement responses of female red deer (*Cervus elaphus*) to drive hunts

**DOI:** 10.1371/journal.pone.0228865

**Published:** 2020-03-09

**Authors:** Agathe Chassagneux, Clément Calenge, Pascal Marchand, Emmanuelle Richard, Etienne Guillaumat, Eric Baubet, Sonia Saïd

**Affiliations:** 1 Direction de la Recherche et de l’Appui Scientifique-Unité Ongulés Sauvages, Office Français de la Biodiversité, Birieux, France; 2 Direction de la Recherche et de l’Appui Scientifique-Unité Flore et Végétation, Office Français de la Biodiversité, Birieux, France; 3 Direction surveillance, évaluation, données-Unité données et appui méthodologique, Office Français de la Biodiversité, Le Perray en Yvelines, France; 4 Pôle Nature, Fondation François Sommer, Paris, France; 5 Direction de la chasse et de la forêt, Domaine National de Chambord, Chambord, France; Institute of Animal Science, CZECH REPUBLIC

## Abstract

Hunting can be used as a tool for wildlife management, through limitation of population densities and dissuading game from using sensitive areas. The success of these approaches requires in depth knowledge of prey movement. Indeed, movement decisions of game during hunting may affect the killing success of hunters as well as the subsequent location of surviving animals. We thus investigated red deer movement responses to drive hunts and their causal factors. We studied 34 hunting events in the National Estate of Chambord (France) and thereby provided a fine-scale characterization of the immediate and delayed movement responses of red deer to drive hunts. Red deer responded to drive hunts either by immediately fleeing the hunted area, or by initially remaining before ultimately fleeing after the hunters had departed. A few hours after the hunt, all individuals were located in distant areas (> 2 kilometres) from the hunted area. Immediate flight responses were less common when drive hunts occurred in areas with dense understorey. However, neither beater/dog densities nor site familiarity influenced the immediate flight decision. Following a drive hunt, red deer remained outside the hunted areas for periods twice as long compared to periods when no hunting occurred (34 hours vs. 17 hours). Such knowledge of game movement rates in response to drive hunts may help the development of informed management policy for hunted red deer populations.

## Introduction

The encroachment of human activities into wildlife habitats has provoked extensive demographic and distributional changes in several species [1,2]. For example, over the last decades, several hunted species in the northern hemisphere have benefited from new hunting regulations and reintroduction programs, or from the low abundance of natural predators; as a result, they have increased steadily and recovered in areas where they had disappeared [3–5]. These changes have led to local damage and an increase in concerns related to security, public health, and economic consideration related to wildlife management [6–12]. In this context, the management of certain large herbivore populations is a growing challenge.

Nowadays, management of hunted ungulate populations in the northern hemisphere almost exclusively relies on limiting population abundance through harvesting plans. However, in a similar way to natural predators, human hunting has consequences not only on prey abundance through direct lethal effects, but also on their behaviour, *i*.*e*. through indirect non-lethal effects [13–17]. Therefore, more recently, reducing the negative impacts of ungulates on habitats and human activities through the use of non-lethal hunting effects has been proposed as a new management tool [11,18,19]. Human hunting generally leads to animals avoiding hunted areas at short or longer time scales (*e*.*g*. by fleeing, using non-hunted or protected areas more intensively, or increasing nocturnal activity; [20–22]). By focusing hunting on sensitive areas (*e*.*g*. croplands or forest regeneration areas) during periods of potential damage, and by creating a heterogeneous spatio-temporal ‘landscape of fear’, managers may thus limit damage by forcing animals to relocate to less sensitive areas [11]. Yet, little is known about the spatial and temporal extent of movement following hunting: how far from the hunted area prey flee–if they do–and for how long?

When prey are directly exposed to a predation threat, two movement responses are generally observed: either the prey flees or it stays and tries to hide (natural predation: [23–25], human predation: [26–28]). However, these movement responses are generally described over short periods of time (*e*.*g*. from the encounter with the predator to a few hours later [28]), while very few studies have investigated movement responses more globally by considering their spatial and temporal dynamics (but see [29]). Other movement responses of prey to predation threat, such as delayed flight after the departure of predators, have been widely reported (natural predation: [24], human predation: [26,29–33]). This questions the simplistic dichotomous view of an immediate flight/remain response of prey to predators, and suggests a more complex behaviour occurring over a longer period of time. A more global description of these movements would help to achieve a better understanding of this movement response.

In addition, little is known about the factors shaping animal movement after hunting. However, in the context of natural predation, it has been shown that such movement responses result from the ability of prey to assess threats, to balance the risk of predation and the benefits resulting from any other activity, and eventually to choose appropriately between alternative anti-predator responses [13,34]. The role of habitat features on the perception of predation risk and the resulting anti-predator responses of prey have already been documented for a wide range of species [24,35–37]. These studies highlighted that the perceived predation risk increases with habitat openness, which is related to a decrease in the density of protective vegetation cover, or to an increase in the distance to refuge areas [24,35–39]. A good knowledge of areas where encounters with predators occur is also suspected to confer an advantage to prey in predator-prey interactions, as revealed by some studies. For instance, Snyder *et al*. [40] demonstrated a high level of predation on mice (*Mus musculus*) when they had little knowledge of their environment. Similarly, Clarke *et al*. [41] showed that the escape tactics used by chipmunks (*Tamias striatus*) varied depending on whether they were approached by humans within their home range or outside of it.

Both the occurrence of an encounter between a prey and its predators, and the prey’s decision to flee immediately or not, may also depend on the predator’s characteristics (*e*.*g*. speed, directness, density, detection capacities; [37]). For example, the number of predators has been shown to strongly affect flight initiation in Thomson’s gazelles (*Eudorcas thomsonii*), with smaller distances travelled when they encounter a single predator rather than a group of predators [42]. As far as we know, habitat features of the area where the prey-predator encounter occurs, knowledge of the hunted area by the prey, and hunting effort have never been fully considered in studies investigating the effects of human predation on wildlife behaviour.

Previous studies investigating the non-lethal effects of hunting have generally assessed changes in prey spatial behaviour with a strong focus on the animal itself. For instance, they reported flight behaviour when the animal suddenly moved long distances (> 300 meters) after an encounter with hunters [28] or when the animal reached the limits of its home range [29]. Similarly, in a previous study carried out in another study area, Chassagneux et al. [43] described how red deer adjusted their movement rates over several days after hunters left compared to their baseline behaviour. However, to our knowledge, how prey use the hunted area both during and following the encounter with hunters is still poorly documented. Therefore, in this study, we investigated movement responses of 14 adult female red deer to drive hunts, which are the most common hunting technique for this species in France [3], and the most effective one to reduce densities in Europe [10]. Using GPS data collected in the National Estate of Chambord (NEC; France), we studied their movement relative to the hunted area, both during the immediate phase when hunting was taking place, and during the delayed phase after hunting had finished. Based on the risk-disturbance hypothesis, red deer were expected to respond to human predation just as they would to natural predators [14]. We expected hunted red deer to move away from the hunted area (perceived as unsafe) in response to drive hunts. However, we also expected the tendency of red deer to flee away from the hunted area to vary with the perceived risk in the hunted area. In other words, we expected stronger avoidance when the number of gunshots and the number of beaters and dogs was higher [37]. In contrast, we predicted that red deer would preferentially stay within the hunted area and hide to avoid detection by hunters (i) if there was a high density of understorey vegetation to hide within the hunted area [27,35–38] and/or (ii) if they had good knowledge of the given area, so that they had better knowledge of potential refuges [41]. Finally, we hypothesized that the tendency of red deer to return to a given hunted area after fleeing it–and the elapsed time before they did so–would also depend on the number of gunshots, the number of beaters and dogs, and the level of site familiarity (*i*.*e*. its location within the annual home range of the animal). We predicted that hunting would increase the elapsed time before an animal returned to a given area compared to when no hunting occurred, due to temporary avoidance of the hunted area which is perceived as unsafe [26,29,30]. In addition, we expected a longer return time if hunting occurred in a less familiar area (*i*.*e*. the periphery of the annual home range of the animal). By focusing on flight movements from the hunted area and return times, we aimed in this study to document the immediate and delayed responses of red deer to drive hunting, as well as their determinants. This provided insights on the potential contribution of this harvesting technique towards a better management of hunted red deer populations.

## Material and methods

### Ethics statement

The research program is hosted by the NEC and managed by the French Office for Biodiversity (Office Français de la Biodiversité). Both institutions have granted all consents necessary for the fieldwork. Game captures were conducted in accordance with European and French laws. The experiment was designed to minimize animal stress and handling time, and to ensure animal welfare, as defined in the guidelines for the ethical use of animals in research. A specific accreditation was also delivered for capturing animals for scientific and wildlife management purposes. Animal captures and experimental procedures were in line with the French Environmental Code (Art. R421-15 to 421–31 and R422-92 to 422-94-1) and duly approved by legislation from the Prefecture of Paris (Prefectural Decree No. 2013–118, license number 2014178–0009).

### Study site and data collection

**Fig 1:**
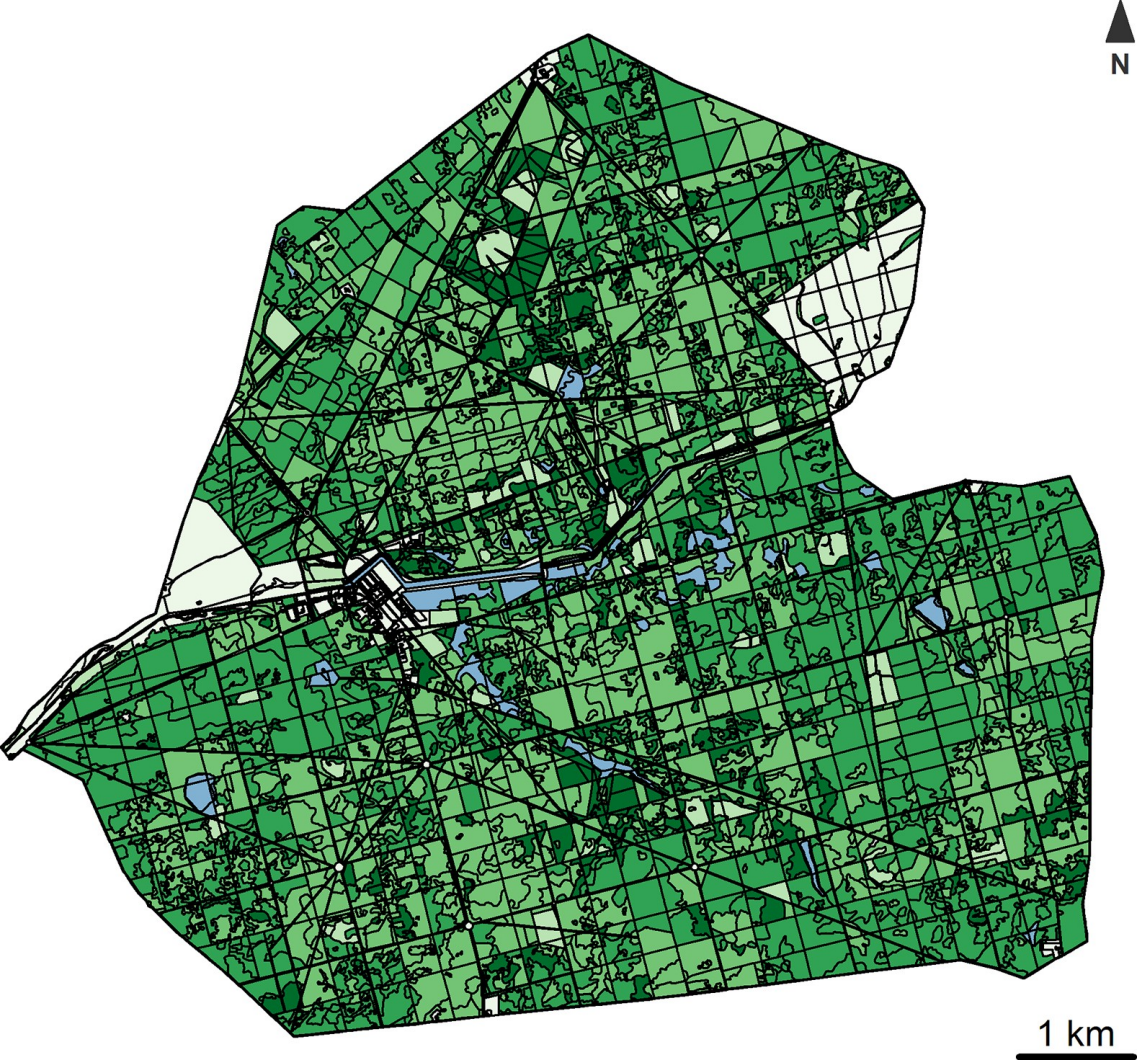
Map of the National Estate of Chambord. Four vegetation classes are shown here: dense understorey areas (in dark green), forest with a sparse understorey, forest without understorey and meadows (in light green). Artificialized areas (*e*.*g*. built-up surfaces or car park) are in light grey and wetlands (ponds, rivers and wet areas) are in blue. This figure was drawn using the R package sf [44].

The study was conducted in the NEC (47°36 N, 1°31 E, France), a 5439 ha wall-fenced park located at low elevation (72–128 meters above sea level). The size of the park is large enough so that the movements of animals is not impeded (*i*.*e*. ten times larger than the annual home range of the monitored animals; mean = 561.1 ha, SD = 282.5 ha; ~10% of the size of the park). Yet, one can consider that the fence of the study area may affect the responses of animals to drive hunts, similarly to many natural or artificial features usually encountered in nature (*e*.*g*. rivers, cliffs or roads). However, we checked whether the animals which were disturbed close to the wall consistently exhibited only one of the two immediate responses we observed (either fleeing or staying during the drive hunt). The two behaviours were both adopted by animals located close to the wall at the time of the drive hunt (see S1 Fig for further details). In the study area, the climate is semi-oceanic with cool summers and mild winters (minimum and maximum temperatures in January: [1.7°C– 8.5°C] and July: [13.1°C– 27.5°C], mean total annual rainfall of 665.7 mm, data from Romorantin weather station, Météo France, from 2016 to 2018). The forest area lies on acidic sandy soils and is dominated by oak (*Quercus petraea* and *Quercus robur*) and coniferous trees (mostly *Pinus sylvestris*). We characterized vegetation cover in the hunted areas using both geo-referenced aerial photographs and field observations conducted over the entire fenced area (last map update in 2015; Fig 1). We distinguished dense understorey areas, which may provide shelter during the drive hunts, from all the other types of vegetation cover. Dense understorey included areas dominated by brambles (*Rubus fructicosus*), bracken ferns (*Pteridium aquilinum*) and broom (*Cytisus scoparius*).

Four ungulate species were present in the NEC and managed through hunting: wild boar (*Sus scrofa*; average number of individuals harvested per year (2016–2018): 941 individuals, range = [900–981]), red deer (*Cervus elaphus*; average number of individuals harvested per year (2016–2018): 214 individuals, range = [200–227]), roe deer (*Capreolus capreolus*; average number of individuals harvested per year (2016–2018): 3 individuals, range = [2–4]) and mouflon (*Ovis gmelini musimon* x *Ovis* sp.; average number of individuals harvested per year (2016–2018): 28 individuals, range = [21–34]). Drive hunts, the harvesting technique used for 64.2% (2017–2018) of harvested female red deer, occurred once a week (every Friday, from approximately 9 a.m. to 6 p.m.) from mid-November to the end of February, with 4 to 6 drive hunts per day (a drive hunt usually lasts about 60 minutes). Each hunting day, 36 beaters (range = [min: 30-max: 45]) and 40 short-legged dogs (range = [30–50]) (*e*.*g*. fox terrier, beagle) flushed out the game located within the hunted area, *i*.*e*. on average 101 ha (range = [47–201]), bounded by 33 shooters (range = [36–43]) generally hiding in designated hunting stands distributed at regular intervals around the hunted area. A given area could be hunted 1–5 times per hunting season.

In January-February 2017, we captured 14 adult female red deer using drive nets and equipped them with GPS collars (WildCell MG, Lotek Wireless, Ontario, Canada). We then monitored all captured individuals for one year. In order to investigate the movement responses of red deer to drive hunts in detail, we programmed the collars to record one relocation every other minute from 8 a.m. to 8 p.m. each hunting day, and every hour during the other days from mid-November to February, *i*.*e*. the period when drive hunts occurred. The mean fix success rate of the GPS collars was 82% (± 12%) when recording one relocation every other minute, and 86% (± 8%) when recording one relocation per hour. The GPS location error δ from BBMM was estimated as 5.8 m (0.05 and 0.95 quantiles: 1.6 and 17 m, respectively [45]). Hunters were requested not to kill GPS-collared individuals during the entire hunting season. In addition, hunted areas were chosen by the NEC independently of the position of GPS-collared red deer.

## Statistical analysis

### Characterizing immediate and delayed movement responses of red deer to drive hunts

We defined a hunting event (n = 34 during the 23 hunting days and based on 14 different individuals monitored with GPS collars) as the presence of a monitored individual deer within the limits of the hunted area during a drive hunt. For each hunting event, we calculated three movement metrics to describe red deer movement paths during the drive hunt: maximum speed, trajectory sinuosity (calculated as the mean cosine of the turning angles; the value 0 corresponds to a Brownian type motion, while the value 1 represents a straight-line movement) and the total cumulative distance covered during the drive hunt (by summing the two-minute step lengths recorded between the start and the end of the drive hunt). The total cumulative distance covered by each animal at the subsequent dawn following the hunt (*i*.*e*. at 8 am) was also computed to describe red deer movement paths following hunting disturbance (by summing the one-hour step lengths recorded between the end of the drive hunt and the next dawn).

We then classified each hunting event into one of the two possible immediate phases of each deer’s response to drive hunts: either fleeing outside the hunted area (*i*.*e*. by crossing the shooting line that delimited the hunted area) or remaining within it as the risk of being killed during the hunt mostly depends on whether the individual crosses the shooting line or not. As the movement response of the animal to the hunt may also be expected to last after the hunting event, *i*.*e*. a delayed phase, we also compared net displacement between the deer’s position inside the hunted area at the beginning of the drive hunt and locations collected both before (*i*.*e*. two control days) and after (*i*.*e*. two test days) the onset of the drive hunt. This allowed us to describe the spatio-temporal dynamics of the movement responses over the 5 days around each hunting event, distinguishing day and night periods (*i*.*e*. from 8 am to 6 pm, and from 6 pm to 8 am, respectively) within these 5 days.

### Determinants of immediate and delayed movement responses of red deer to drive hunts

**Fig 2:**
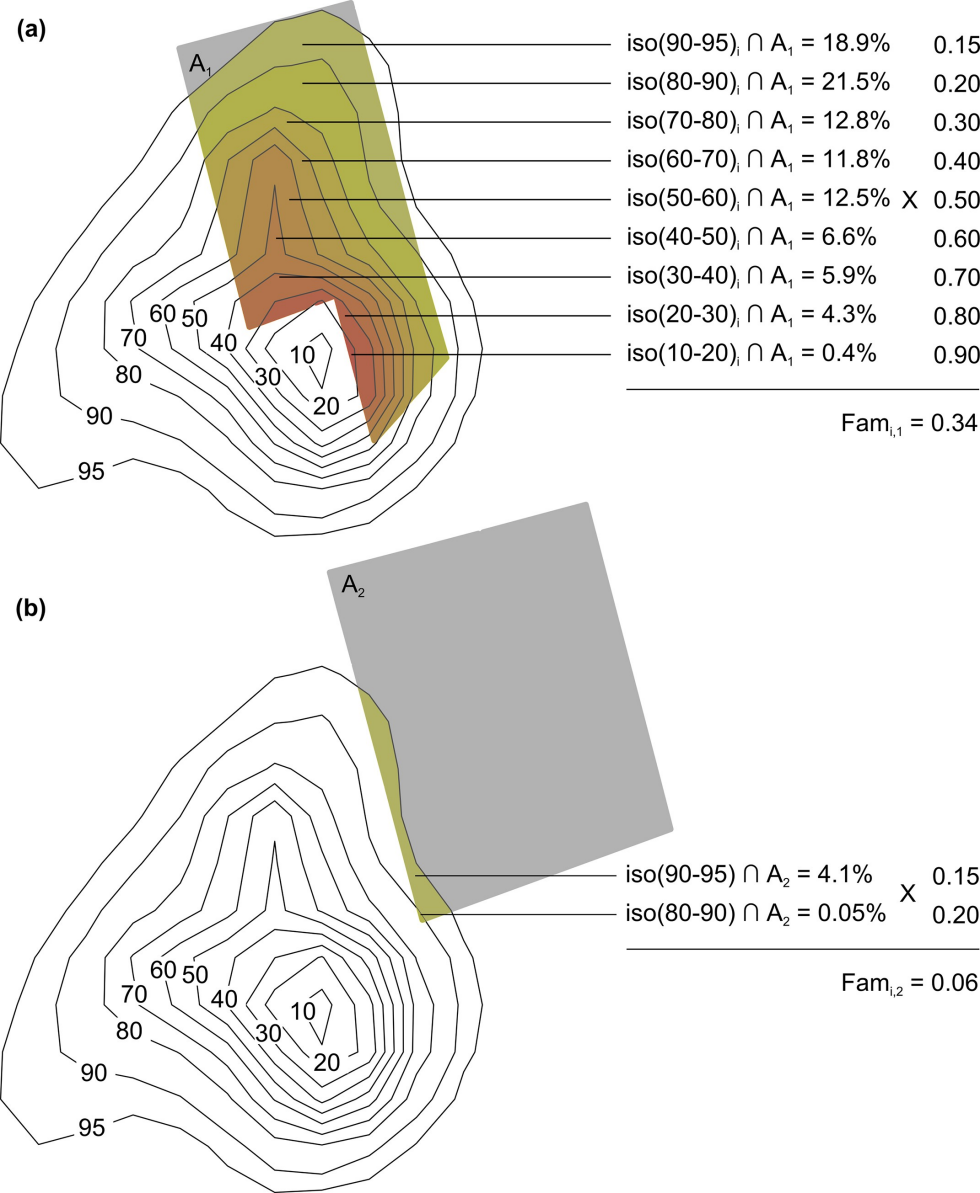
Example of the familiarity index of two hunted areas (A_1_ and A_2_) for the animal *i*. Each polygon corresponds to UD values at a given probability level (from 10 to 95%). The intersections between the area delineated by two successive isopleths and the hunted area (coloured areas, expressed as the percentage of the whole hunted area) are multiplied by a coefficient. The familiarity index (Fam_i,j_) is the sum of all these products.

We investigated the immediate and delayed movement responses of red deer by calculating cumulative distances covered by individuals: (i) during the drive hunt (*i*.*e*. immediate phase), by summing displacement distances during the two-minute step intervals between the start and the end of the drive hunt and (ii) up to the first sunrise following the drive hunt (*i*.*e*. delayed phase), by summing displacement distances during the one-hour step intervals between the start of the drive hunt and the following sunrise, for all hunting events. We then used linear mixed models to assess the effects of habitat characteristics (*i*.*e*. proportion of the hunted area covered by dense understorey; understorey density, named Bush in the model; Table 1), familiarity index of the hunted area (Fam in the model; Table 1), and the hunting disturbance intensity in terms of the densities of dogs and beaters (Dogs and Beat in the model; Table 1) and the number of gunshots fired during the drive hunt (Shot in the model; Table 1) on red deer movement responses. To define the familiarity index of a given hunted area, we first calculated the annual home range of the animal (*i*.*e*. using the relocations collected during the 12 months following the day of capture) as its 95% annual utilization distribution (UD) based on one location every 12 hours and using the fixed kernel method [46]. Then, we split the annual home range of each individual into 10 regions characterized by a decreasing probability of use by the individual, based on the isopleths of the individual’s UD (*e*.*g*. the 95% home range is defined by the isopleth delimiting the minimum surface area where the probability to find the animal is equal to 0.95; Fig 2). In other words, we defined 10 regions ***iso*(*x*_1_−*x*_2_)** (with x_1_ and x_2_ taking values between 0 and 90 and 10 and 95, respectively), each region corresponding to the area of the home range comprised between the UD isopleth x_1_ and x_2_. We then computed the familiarity index of a given hunted area using the following formula:

$${Fam}_{i,j}={iso(0-10)}_{i}\cap j*1+{iso(10-20)}_{i}\cap j*.9+\cdots +{iso(90-95)}_{i}\cap j*.15$$ (1)

where *Fam*_*i*,*j*_ is the familiarity index of the hunted area *j* for the animal *i* and *iso(x*_*1*_ *–x*_*2*_*)*_*i*_ *∩ j* is the intersection between the area comprised between two successive isopleths x_1_ and x_2_ and the hunted area *j* (expressed as the percentage of the whole hunted area).

**Table 1:** Median values and range of the five movement variables used in the model described in Eq 2 and calculated to investigate the determinants of immediate movement responses of red deer in the National Estate of Chambord, France, 2016–2018. Median values and ranges were calculated for all the hunting events (n = 34).

Movement variable	Acronym	Description	Median	Range [minimum–maximum]
Understorey density (%)	Bush	Proportion of the hunted area covered by dense vegetation	6.4	3.6–45.9
Number of gunshots	Shot	Number of gunshots during the drive hunt	40	10–85
Dog density	Dogs	Number of dogs per hunted hectare	0.32	0.21–0.88
Beater density	Beat	Number of beaters per hunted hectare	0.32	0.20–0.76
Site familiarity	Fam	Familiarity index	0.25	0–0.82

Therefore, the familiarity index equals 0 when the hunted area is located outside the annual home range of the animal and 1 when the hunted area is located within the (0–10) isopleth (*i*.*e*. the animal is very familiar with the hunted area; Fig 2). A summarized overview of the four other variables used in the model is given in Table 1. The home range estimation was carried out with the R package adehabitatHR [47].

We fitted the model including the five previously described factors and a random effect corresponding to the ID of the animal (as a given animal may be exposed to several hunting events) as follows:

$$\log\left({\mathrm{Dist}}_{i}\right)=\mu +\alpha .{\mathrm{Bush}}_{i}+\beta .{\mathrm{Fam}}_{i}+\gamma .{\mathrm{Dogs}}_{i}+\delta .{\mathrm{Beat}}_{i}+\rho .{\mathrm{Shot}}_{i}+u_{i}+\varepsilon_{i}$$ (2)

where *log(Dist*_*i*_*)* is the logarithm of the variable of interest (the total cumulative distances covered by individuals during either the immediate or the delayed phase) for the animal *i*; *X*_*i*_ describes the value of a variable *X* (which can be either *Bush*, *Fam*, *Dogs*, *Beat* or *Shot*) for the hunting event involving animal *i*; *α*, *β*, *γ*, *δ* and *ρ* are the coefficients of the explanatory variables (*Bush*, *Fam*, *Dogs*, *Beat* and *Shot* respectively), μ is the intercept of the model; *u*_*i*_ is the random intercept describing within-individual variation with a Gaussian distribution $u_{i}∼N(0,\sigma_{u}^{2})$; and *ε*_*i*_ is the Gaussian residual of the model. We fitted this model within a Bayesian framework using the R package rstanarm [48]. For each parameter, we used the default weakly informative prior distributions [49]. The posterior distributions of the model parameters were thus sampled using a Markov Chain Monte Carlo algorithm (MCMC).

### Return times to the hunted area

We compared the curves representing the probability of returning to a hunted area (1) after a drive hunt and (2) in undisturbed situations (*i*.*e*. before the drive hunt in the same area) using Kaplan-Meier estimates ([50], package survival, [51]). The Kaplan-Meier approach is generally used to estimate the probability of occurrence of an event (usually, the death of an individual or the failure of a unit) as a function of time. Here, we defined the event of interest as the return of an individual to the limits of the focal hunted area. Since the home range of a red deer is much larger than the size of any hunted area in the NEC (see methods), the ultimate departure of any animal from any area of the size of a hunted area is expected whether hunted or not, so that return times can be calculated both with or without hunting. For each hunting event, two return times/censored values were computed: (1) from the flight of the animal as a result of the drive hunt until its return (event of interest) and (2) a control value, obtained by measuring the time lapse between the last time the animal left this area and the time when it came back to the area just prior to the drive hunt. In this latter case, we worked on a reversed time scale, starting at the beginning of the drive hunt (when the animal was in the area) and stopping when the animal last left this area prior to the hunt. If the animal was not present in this area 6 days before the drive hunt, we reported a censored value [50] in order to avoid the overlap between two hunting days, as hunting occurred once a week.

The distribution of return times was clearly bimodal, with some animals returning to the hunted area within 72 hours, and others returning much later. We therefore defined a new variable describing these short return times, with return times shorter than 72 hours coded as 0, and return times longer than 72 hours coded as 1. We then tested the effects of hunting occurrence, familiarity index of the area and their interaction on the probability of a short return time to the hunted area using a generalized linear mixed model with a logit link and a binomial error distribution. We accounted for within-individual variation by including an individual effect as a random factor in the model. Thus, we fitted the following model:

$$\mathrm{logi}t\left(P({Short\;return\;times}_{i})\right)=\mu +\alpha .{Hunt}_{i}+\beta .{Fam}_{i}+\gamma .{Hunt}_{i}.{Fam}_{i}+u_{i}+\varepsilon_{i}$$ (3)

where *logit(P(Short return times*_*i*_*))* is the logit of the probability of a short return time to the hunted area for the animal *i* taking values 0 or 1; *X*_*i*_ describes the value of a variable *X* (which can be either *Hunt* or *Fam*) for the hunting event involving animal *i*; *α*, *β* and *γ* are the coefficients of the explanatory variables (*Hunt*, *Fam* and the interaction between *Hunt* and *Fam* respectively); *μ* is the intercept of the model; *u*_*i*_ is the random intercept describing within-individual variation with a Gaussian distribution ***Δ***; and *ε*_*i*_ is the Gaussian residual of the model. We also fitted this model within a Bayesian framework using the R package rstanarm [48]. For each parameter, we used the default weakly informative prior distributions [49]. The posterior distributions of the model parameters were thus sampled using a Markov Chain Monte Carlo algorithm (MCMC). We performed all these analyses using the R version 3.4.2 [52].

## Results

### Immediate and delayed movement responses of red deer to drive hunts

**Fig 3:**
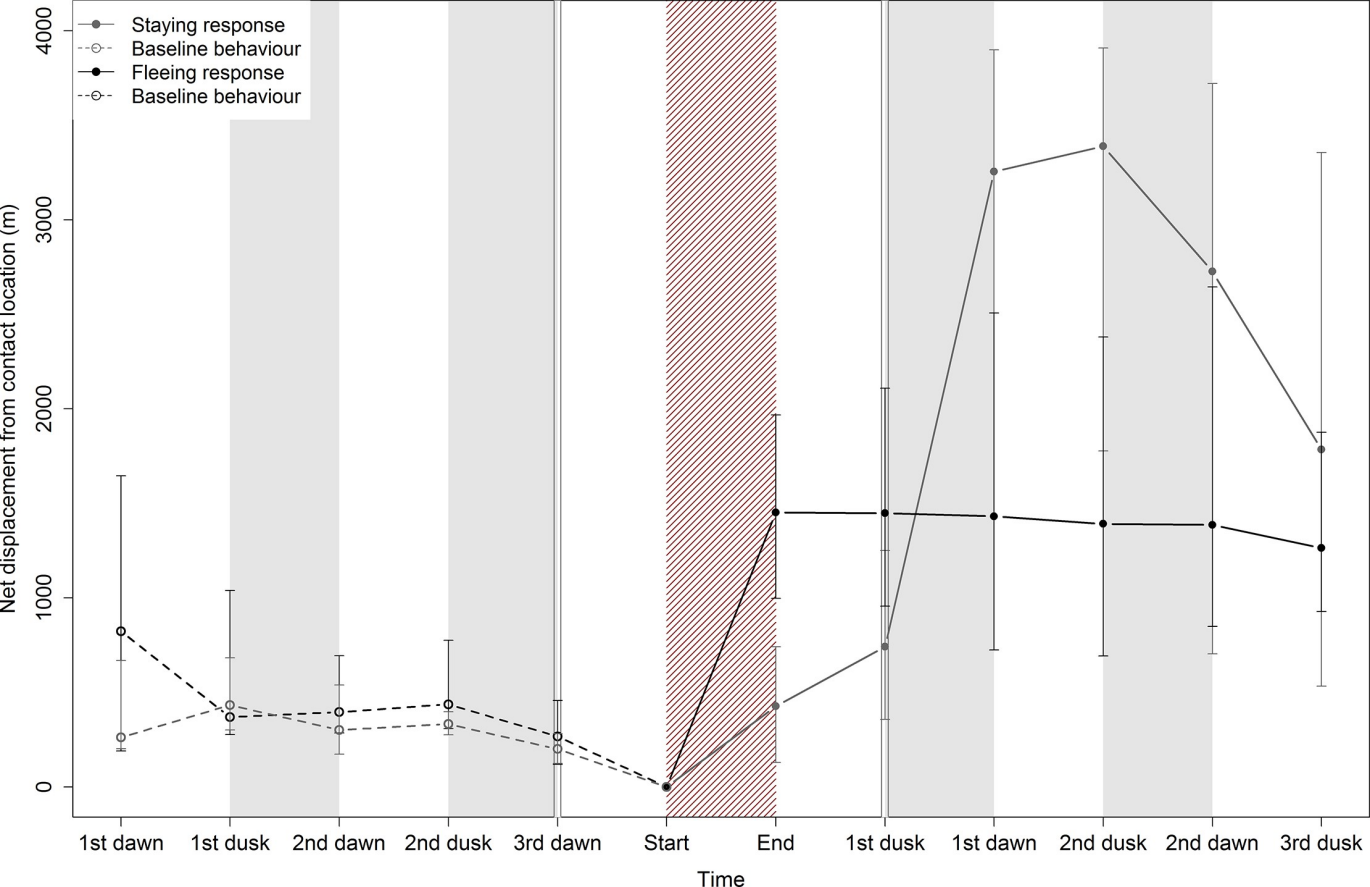
Median net displacement of red deer from the encounter site with hunters (*i*.*e*. distance from the location of the monitored individual at the beginning of the drive hunt) calculated over a five-day period in the National Estate of Chambord, France, 2016–2018. Two responses were distinguished during the immediate phase based on whether or not the individual crossed the shooting line and moved out of the hunted area during the drive hunt. The black line describes movement patterns of red deer ‘fleeing’ from the encounter site (n = 23), whereas the grey line describes these patterns for ‘remaining’ individuals (n = 12). Black dots correspond to responses following a contact with hunters, whereas white dots correspond to the baseline behaviour (*i*.*e*. during the hunting season, but in the absence of hunters) of individuals. First and third quartiles are represented using vertical bars. The grey areas represent the night-time period. The red hatched area corresponds to the drive hunt period. The plot was divided into three panels in order to illustrate the non-continuous time scale: (1) one relocation every hour from 1^st^ dawn to 3^rd^ dawn, (2) one relocation every other minute from 3^rd^ dawn to 1^st^ dusk and (3) one relocation every hour from 1^st^ dusk to 3^rd^ dusk.

In the present study, red deer responses to hunting were investigated as a continuous response, but by distinguishing two phases: (i) the immediate phase at the time of the drive hunt and (ii) the delayed phase after the departure of hunters. In our study site, red deer exhibited flight behaviours during the immediate phase of the response in 68% of the hunting events, while in the remaining 32%, they remained in the hunted area. Total cumulative distances covered by ‘fleeing’ red deer were significantly longer than those of the ‘remaining’ red deer at the end of the drive hunt (Student test, t = -4.84, df = 32, p < 0.001). During the drive hunt, the ‘fleeing’ red deer showed a long cumulative distance travelled, a high maximum speed, a straighter movement (Table 2) and a long net displacement from the encounter site (median: 1452 m [424.2–3535]; fleeing phase at the end of the drive hunt; Fig 3). Conversely, ‘staying’ red deer covered a total cumulative distance of less than 1000 metres, at a maximum speed of about 10 km.h^-1^, along a tortuous path with many direction changes (Table 2) and moved 426.3 m [5.05–1227] away from the encounter site (staying phase at the end of the drive hunt; Fig 3).

**Table 2:** Median values and range of the four movement metrics used to describe movement paths of red deer in the National Estate of Chambord, France, 2016–2018. All these metrics were obtained using data from GPS collars (based on one location every other minute for the first three and one location every hour for the fourth). Values were then calculated for the two groups of individuals: (a) ‘staying’ (32%) and (b) ‘fleeing’ (68%) discriminated based on the immediate phase of their response (*i*.*e*. whether the individual crossed the shooting line and moved out of the hunted area during the drive hunt or not).

Movement variable	Acronym	Description	Groups	Median	Range [minimum–maximum]
Maximum speed (km.h^-1^)	Speed	Maximum speed of the two-minute step lengths recorded between the start and the end of the drive hunt	‘Staying’	9.70	0.60–20.9
			‘Fleeing	18.0	8.23–28.5
Mean sinuosity	Sin	Mean sinuosity of the two-minute step lengths recorded between the start and the end of the drive hunt	‘Staying’	-0.29	-0.46–0.21
			‘Fleeing	0.04	-0.29–0.84
Cumulative distances covered during the drive hunt (m)	DistI	Sum of the two-minute step lengths recorded between the start and the end of the drive hunt	‘Staying’	940.9	170.1–1873
			‘Fleeing	2065	577.5–5175
Cumulative distances covered up to the first sunrise following the drive hunt (m)	DistD	Sum of the one-hour step lengths recorded between the start of the drive hunt and the first following sunrise	‘Staying’	5027	2235–6660
			‘Fleeing	6721	2044–11229

However, by the first dawn after the encounter, all red deer had covered around 5000 metres (fleeing: 6721 m [2044–11229], staying: 5027 m [2235–6660]) with no significant differences between the two groups of individuals (defined based on the immediate phases of their response) in total cumulative distance covered (Student test, t = -1.51, df = 32, p = 0.14). By the first dusk after the drive hunt, all red deer had left the hunted area. After the drive hunt and before the first sunrise, all red deer had moved 2158 m ([1078–3254]; staying and fleeing responses at first dawn; Fig 3) away from the hunted area, *i*.*e*. a distance four times greater than that usually covered in the absence of hunters (395 [238–600]; *i*.*e*. movement behaviours observed before the drive hunt; ‘baseline behaviours’ in Fig 3).

### Determinants of immediate and delayed movement responses

**Fig 4:**
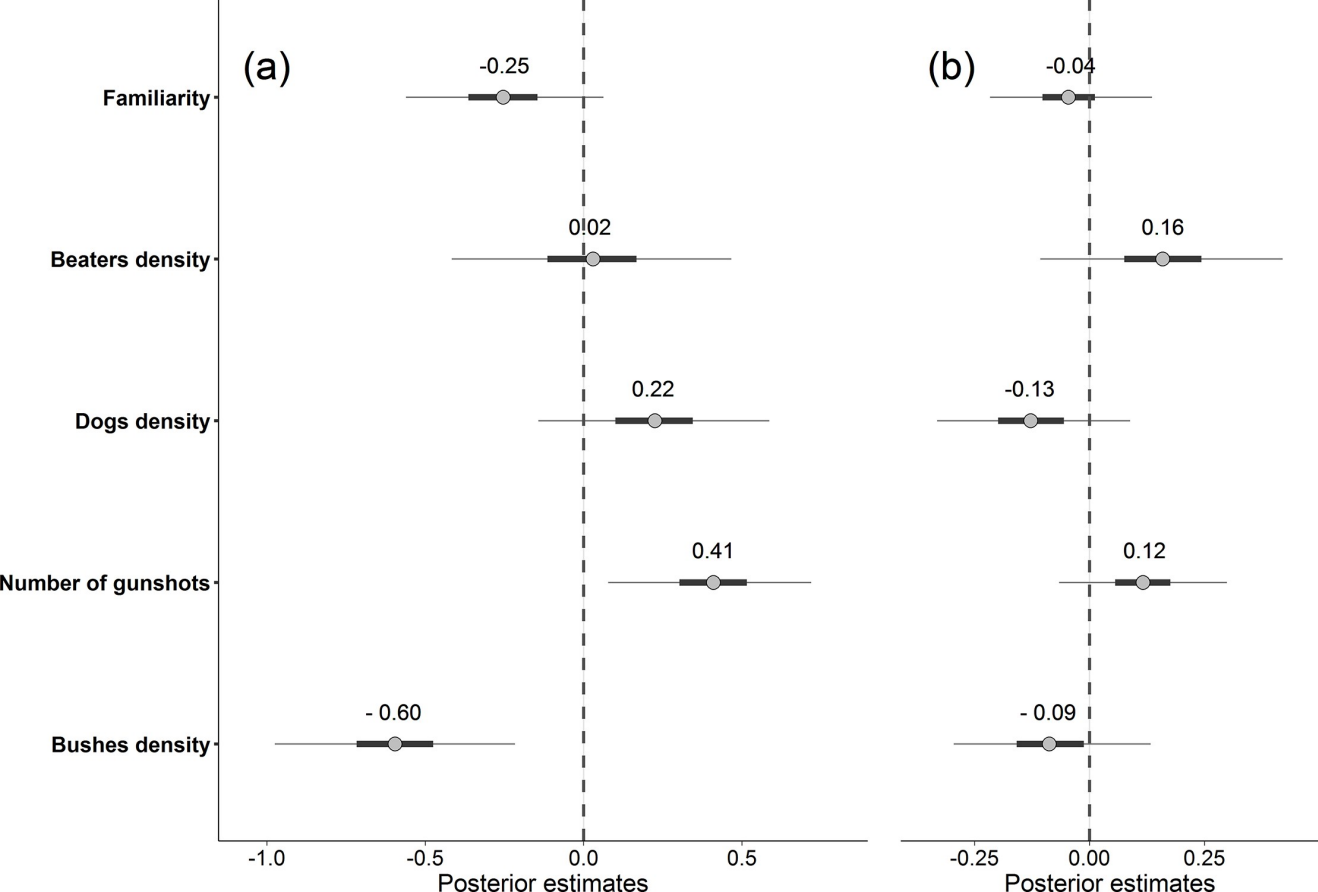
**Estimates of the effects of site familiarity, beater density, dog density, number of gunshots during the drive hunt and understorey density within the hunted area on the distance covered by red deer (a) during the drive hunt and (b) over the 12 hour period following a drive hunt in the National Estate of Chambord, France, 2016–2018.** Grey dots represent posterior medians. Error bars show 95% credible intervals. Error bars identified by the thicker line show 75% credible intervals. Each tested variable had a significant effect on the immediate phase of red deer movement responses when the 95% credible intervals (dashed lines) did not overlap 0.

On one hand, we found that red deer movement responses during the drive hunt (characterized here by the total cumulative distance covered during the drive hunt hour) were mainly influenced by understorey density within the hunted area and the number of gunshots during the drive hunt. Total distance covered by red deer during the drive hunt was negatively correlated with understorey density within the hunted area (Fig 4A). In contrast, the number of gunshots during the drive hunt was positively related to the distance covered by hunted red deer during the drive hunt (Fig 4A). However, we found no support for the hypotheses that risk characteristics of the drive hunt in terms of beater and dog densities or site familiarity affected the distance covered by red deer during the drive hunt (Fig 4A).

On the other hand, we found no support for the effect of these four variables–understorey density, number of gunshots, site familiarity and dog and beater densities–on the total distance covered by red deer during the 12 hour period following the drive hunt (*i*.*e*. the delayed phase of the response; Fig 4B).

### Return times to the hunted area

**Fig 5:**
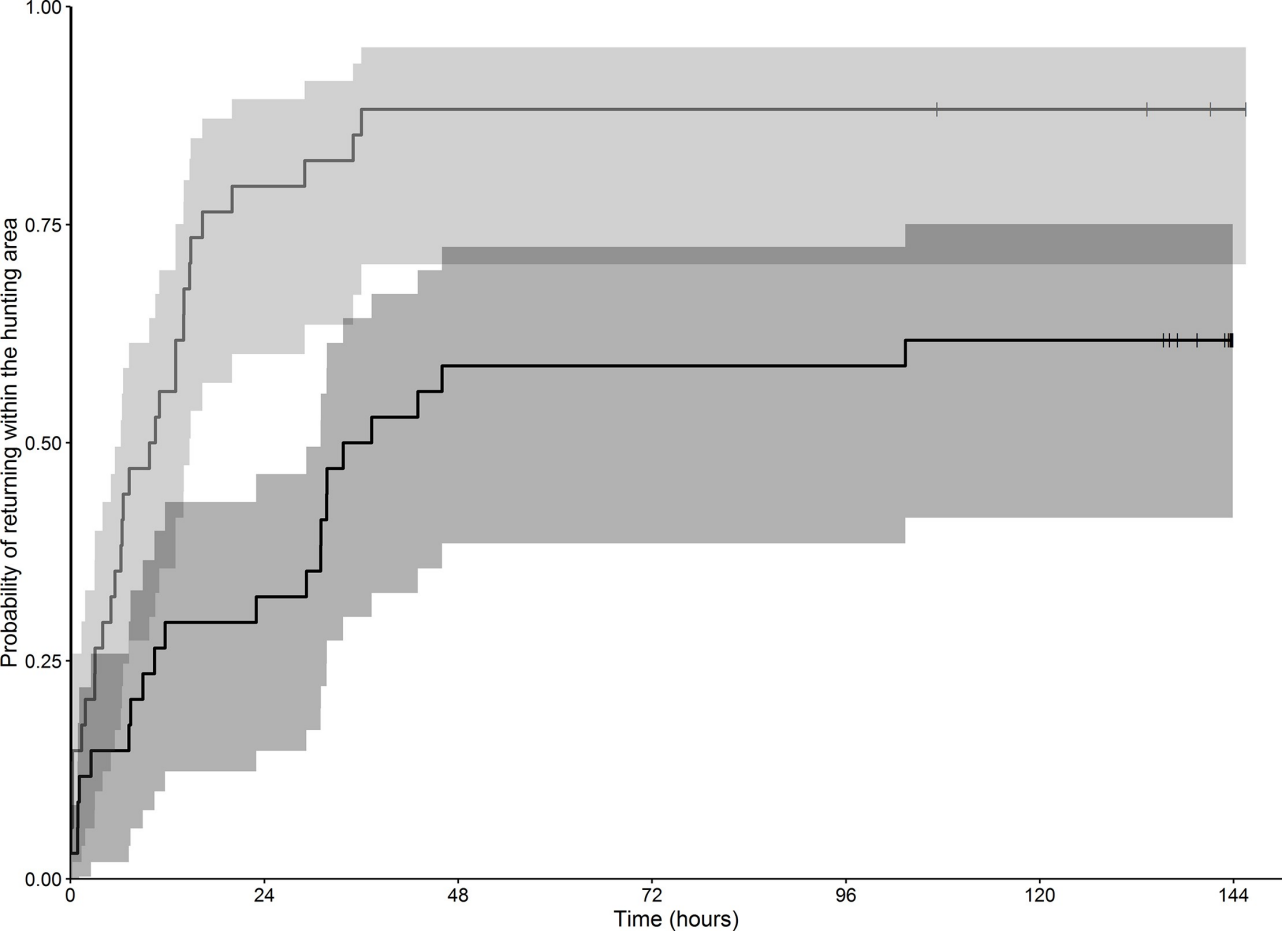
Kaplan-Meier estimates of the probability of returning to the hunted area before (in light grey) and after hunting (in dark grey) as a function of time. Grey parts on both sides of the lines correspond to 95% confidence intervals. Small vertical bars crossing the curves indicate censored values (*i*.*e*. no return to the area, even one week after the drive hunt).

The median return time after hunting was 34 hours, *i*.*e*. twice as long as the median return time with no hunting (17 hours). In addition, 13 of the 34 individuals did not return to the hunted area over the 6-day period following a drive hunt, while only four individuals did this in the absence of hunting. Furthermore, the Kaplan-Meier estimates revealed that red deer came back faster to a given area in the absence of any hunting disturbance than after having experienced a drive hunt at this place (Fig 5).

The generalized linear mixed model on red deer that actually returned to hunted areas within 6 days showed that the probability of a short return time was affected by both hunting disturbance and the familiarity index: red deer came back more slowly to the hunted area (i) when they had experienced a drive hunt (positive coefficient of the effect of the drive hunt on the probability of a short return time; median = 1.8, 95% CI = [0.22, 3.54]) and (ii) when the hunted area was less familiar to the animal (negative coefficient of familiarity on the probability of short return time; median = -5.3, 95% CI = [-12, -0.09]). However, we could not identify any strong effect of the interaction between drive hunt occurrence and familiarity on the probability of a short return time (median = 0.64, 95% CI = [-6.0, 7.8]).

## Discussion

Our study provides a fine-scale characterization of the spatio-temporal dynamics of red deer responses to human hunting using precise knowledge of game locations. We focused here on describing the outgoing movements of red deer from the hunted area, as well as the time lapse before returning, in order to serve as a framework for management actions of hunted red deer populations. We highlighted that the immediate decision of red deer in response to drive hunts–fleeing or staying–was mainly influenced by vegetation cover within the hunted area, and was only weakly related to the number of gunshots. Regardless of the decisions taken by the animals during the drive hunt, all red deer ultimately left the hunted area and fled towards distant areas. In addition, after hunting, red deer remained away from the source of disturbance for periods that were almost twice as long as those in the absence of any disturbance. We further discussed these results below.

### Immediate movement responses of red deer to drive hunts: a matter of local context

Most female red deer (68%) escaped hunters by leaving the hunted area during the drive hunt. In contrast, other individuals remained hidden in refuges within the hunted area. These responses can be compared to previous findings on natural predators (*e*.*g*. [53]), supporting the risk-disturbance hypothesis [15]. Covering long distances to leave the hunted area may be costly (*e*.*g*. lost feeding opportunity) but may allow to fully eluding the hunters, while remaining hidden may increase the risk of being captured if available refuges are not safe enough, as prey may lose visual contact with the hunters. In addition, in the context of human hunting, beaters and dogs are usually used to flush out wild game hiding in refuges. This might force animals to move and minimize the number of animals that remain hidden within the hunted area. Therefore, the choice between the two responses may be dependent on the microhabitat cover, the knowledge of the potential surrounding refuges or the risk posed by the hunters.

Our results showed that red deer had greater movement rates during the drive hunt when they were confronted with hunters (*i*.*e*. beaters or shooters) in an area with less dense understorey. Similar to red deer, beaters and dogs may have difficulty moving in dense vegetation, thus decreasing the probability of animals being detected and then killed by shooters during the drive hunt. Areas of dense vegetation may hence provide potential refuge areas where red deer might shelter. This result is consistent with previous studies of flight decisions in more open habitats or when the distance to refuge is higher [35–38], presumably because fleeing is more successful than the ‘staying’ tactic where vegetation is less dense. In addition, in a previous study on a similar red deer-hunter system, it has been shown that the absence of dense vegetation also lead to an increase in red deer speed during the three days following the drive hunt [43]. All these studies tend to support the hypothesis that bushy areas impact the effect of drive hunts on the movements of hunted animals. Moreover, regarding site characteristics of the encounter area, we found little or no evidence that site familiarity affected the immediate decisions of red deer during the drive hunt. Therefore, animals that are very familiar with the hunted area have the same probability to increase their movement rates and reduce detection by the hunters through the use of refuge areas within the hunted area.

Predation risk assessment after the encounter with a predator also depends on the information available to the individual inside the encounter area and requires identifying visual, auditory or olfactory cues of the predator’s presence [54,55]. Deer rely in the first place on smell and sound to detect environmental changes and predators [56,57]. Anthropogenic noise, such as gunshots, is known to have several negative impacts on animals (*e*.*g*. movement pattern changes, increased physiological stress levels, altered communication; [58–61]) and can lead to responses such as flight or avoidance of the source of noise. Our results support this idea, since they reveal that gunshots fired during the drive hunt are correlated with longer distances covered by red deer during drive hunts. In addition to noise, the number of gunshots may also be summarizing proxy for the intensity of hunting disturbance, since it reflects (i) the efficiency of beaters in forcing animals to move towards the shooting line and (ii) the number of animals of other species (wild boar, roe deer or mouflon) leaving the hunted area. In this case, the longer distances covered by red deer could be attributed to more intense drive hunts.

However, we found no support for the hypothesis that the immediate response decision of red deer is conditional on the density of beaters or dogs. One possible reason is the lack of variability in both the number of beaters and dogs from one drive hunt to another. In addition, the role of beaters and dogs might be over-ridden by the effects of refuge density (*i*.*e*. bushy areas) within the encounter area. It has been suggested that the effect of refuge distance may exceed all predatory effects (*e*.*g*. speed, directness or size, [37]). Therefore, this result highlights the need for further comparative research in order to determine whether it could be confirmed at a broader scale.

We then investigated red deer responses after the departure of hunters. Our results suggest that, after the drive hunt, all red deer fled and ultimately left the hunted area. Since the annual home range of a red deer (mean = 561.1 ha, SD = 282.5 ha) is much larger than the size of any hunted area (mean = 101 ha, SD = 30 ha) in the study area, we expected the animals to eventually leave a given area, even in the absence of hunters. However, by comparing the net displacement of animals before and after hunting, we observed that animals that did not flee during the drive hunt still covered very large distances during subsequent movement away from the hunted area following the hunting event (Fig 2). This result suggests delayed flight, probably as a response to the intense disturbance they experienced during the event, as a way to increase the distance between them and the source of disturbance. This risk avoidance strategy has already been documented in both human and natural predation contexts at different spatial and temporal scales. Prey species may either move away from an encounter area [24], spatially or temporally avoid areas with a high risk of encountering a predator [21,62] or use areas with no predators [20].

Ungulate management, through fear, may require adaptive hunting or site management methods adapted to the openness of the vegetation within the hunted area. For example, since more open areas appeared to lead to flight decisions outside of the hunted area during the drive hunt, they may be favoured to increase shooting opportunities and consequently to reduce local population abundance. Alternatively, since a high density of vegetation in the hunted area leads to more static behaviour of deer during the drive hunt, the probability of flushing an animal could be increased by limiting the size of the hunted area (thereby increasing the density of beaters/dogs in the area) or by increasing the number of trips by beaters and dogs inside the hunted area in order to flush out the hidden individuals.

### Long lasting avoidance of the hunted area

Minimizing red deer impacts on sensitive areas (*e*.*g*. forest regeneration areas) also requires a knowledge of the temporal persistence of this ‘remoteness from the hunted area’ phase. In our study area, we found a median return time of 34 hours after hunting, which was almost twice as long as the one prior to hunting (median: 17h). This result is consistent with the average return time of 23h found by Jarnemo and Wikenros [28]. These findings suggest that red deer took longer to come back to a given area after having experienced a drive hunt than in the absence of any hunting disturbance, probably due to an avoidance reaction in response to the stressful experience suffered in the hunted area. Our results also showed that the number of individuals that did not come back within the week between two drive hunts was three times higher after hunting (38% versus 12% before hunting). This advocates for the use of hunting for fear as a management approach, but it highlights the need for further research on ungulate responses to repeated drive hunts in order to understand habituation to disturbance. If regular disturbance exacerbates red deer responses and forces them to move away from danger over longer periods, then it could be possible to suggest optimal drive hunt frequencies. However, although the effects of the level of exposure to human disturbance on prey responses remains poorly documented, a few studies have shown that repeated disturbance and habituation with time or experience might actually lower the intensity of prey responses. Thus, Thurfjell *et al*. [63] suggested that learning from previous hunting experience may shape anti-predator responses. As they aged, female elk appeared to adjust their behaviour and adopt more cautious strategies such as reduced movement rates and increased use of refuge areas [63]. Such findings highlight the need to further study the cumulative effects of drive hunts on ungulate responses in order to improve their management through non-lethal effects.

The effects of drive hunts may appear limited in time for most individuals due to mitigation by other external (*e*.*g*. site familiarity, foraging) or internal (*e*.*g*. personality) factors that may also affect the return time to a disturbed area. We showed a marginally significant negative effect of site knowledge on the probability of a short return time to a specific area. This is expected even in the absence of hunting, as by definition, familiar areas are used more frequently (*i*.*e*. return time is by definition shorter in familiar areas). However, our hypothesis was that the elapsed time before the animal comes back to a specific area could have been affected by an interaction between familiarity and the occurrence of hunting. Indeed, unfamiliar areas are generally associated with a decreased capacity to detect and avoid predators, and with a greater difficulty to find high quality resources [41,64]. Forrester *et al*. [65] even related the use of familiar areas to mortality risk: black-tailed deer (*Odocoileus hemionus columbianus*) with a tendency to leave their home range are more likely to die mostly due to puma (*Puma concolor*) predation. Therefore, rapidly returning to familiar areas, even after disturbance, can confer significant advantages in foraging activities and in predator-prey interactions. However, since we could not find any effect of the interaction between drive hunt occurrence and familiarity on the elapsed time before red deer came back to a specific area, we could not establish a stronger avoidance of a given area because of a drive hunt when the hunted area was less familiar.

## Conclusion

This study has several implications for both the general understanding of the spatio-temporal dynamics of red deer movement responses during and after drive hunts (*i*.*e*. immediate and delayed phases, respectively) and for programs related to ungulate management through hunting. Our results suggest that total cumulative distances covered by prey during the drive hunt are mostly linked to the surrounding environment of the animal and, more specifically, to the density of vegetation within the hunted area, but only marginally explained by hunting disturbance intensity (*i*.*e*. the number of gunshots fired during the drive hunt). Regardless of the drive hunt characteristics, all red deer moved away from the hunted area and remained in distant areas, for periods twice as long as when there was no hunting disturbance. Hunting is currently used as a management tool to reduce ungulate damage through its lethal effects and, more recently, its non-lethal effects. Game management combines economic (*e*.*g*. hunting, forestry and crop productions), environmental (*e*.*g*. preservation of threatened species) and social (*e*.*g*. hunters satisfaction) objectives. Depending on these local objectives, wildlife managers might want to increase killing success of hunters, minimize negative impacts of prey on the local environment and the economic efficiency of human activities [10–12], limit unwanted use of livestock areas and/or avoid impacts on non-targeted threatened species. However, changes in prey movement during and after hunting may clearly affect the success of management through hunting. Indeed, the killing success of hunters directly depends on an increase in prey movements, which may both facilitate their detection [32] and increase shooting opportunities. In addition, animal movement decisions during hunting may determine their use of sensitive areas (*e*.*g*. crops or wildlife-livestock contact areas) during the following days and hence the efficiency of management by fear. Further investigations on the immediate movement responses of animals and their consequences at broader spatio-temporal scales may be of prime importance in this context.

## Supporting information

S1 Fig Hunting events in relation to the fence of the study area. The grey line corresponds to the wall surrounding the study area. Each hunting event is characterized by a black line with a blue point for the animal’s first location at the onset of the drive hunt and a red point for the animal’s last location at the end of the drive hunt. The panel (A) corresponds to the group of individuals defined as ‘fleeing’ individuals based on the immediate phase of their response and the panel (B) corresponds to the ‘staying’ group. The two behaviours are both shown by the animals located close to the wall at the time of the drive hunt.
(DOCX)
