# Supplementary material for: Should I stay or should I go? Determinants of immediate and delayed movement responses of female red deer (Cervus elaphus) to drive hunts
Source: PLoS One. 2020 Mar 9;15(3):e0228865. doi: 10.1371/journal.pone.0228865 (PMC7062277; doi:10.1371/journal.pone.0228865)

# Supporting information

**S1 Fig. Hunting events in relation to the fence of the study area.** The grey line corresponds to the wall surrounding the study area. Each hunting event is characterized by a black line with a blue point for the animal’s first location at the onset of the drive hunt and a red point for the animal’s last location at the end of the drive hunt. The panel (A) corresponds to the group of individuals defined as ‘fleeing’ individuals based on the immediate phase of their response and the panel (B) corresponds to the ‘staying’ group. The two behaviours are both shown by the animals located close to the wall at the time of the drive hunt.


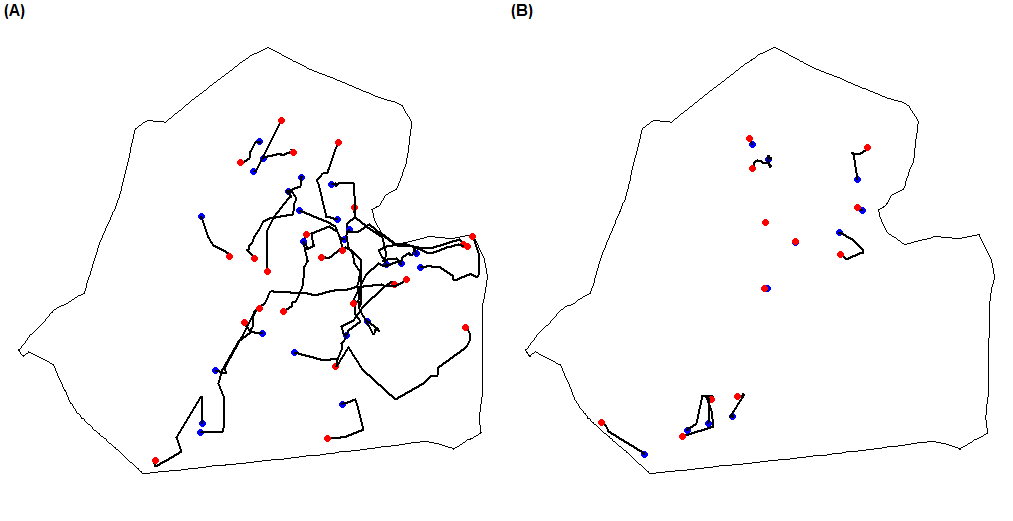

Supplement: S1 Fig — The grey line corresponds to the wall surrounding the study area. Each hunting event is characterized by a black line with a blue point for the animal’s first location at the onset of the drive hunt and a red point for the animal’s last location at the end of the drive hunt. The panel (A) corresponds to the group of individuals defined as ‘fleeing’ individuals based on the immediate phase of their response and the panel (B) corresponds to the ‘staying’ group. The two behaviours are both shown by the animals located close to the wall at the time of the drive hunt. (DOCX) [file pone.0228865.s001.docx]
